# Total En Bloc Spondylectomy for Locally Aggressive Vertebral Hemangioma Causing Neurological Deficits

**DOI:** 10.1155/2015/724364

**Published:** 2015-03-30

**Authors:** Ryo Ogawa, Tomohiro Hikata, Shuji Mikami, Nobuyuki Fujita, Akio Iwanami, Kota Watanabe, Ken Ishii, Masaya Nakamura, Yoshiaki Toyama, Morio Matsumoto

**Affiliations:** ^1^Department of Orthopaedic Surgery, School of Medicine, Keio University, 35 Shinanomachi, Shinjuku, Tokyo 160-8582, Japan; ^2^Division of Diagnostic Pathology, Keio University Hospital, 35 Shinanomachi, Shinjuku, Tokyo 160-8582, Japan; ^3^Department of Advanced Therapy for Spine and Spinal Cord Disorders, School of Medicine, Keio University, 35 Shinanomachi, Shinjuku, Tokyo 160-8582, Japan

## Abstract

Vertebral hemangiomas are common; however, aggressive vertebral hemangiomas with extraosseous extensions causing neurological deficits are rare. The treatment for this subtype of hemangioma remains controversial, since there are few reports on long-term clinical outcomes or tumor recurrence rates. We describe a case of aggressive vertebral hemangioma treated by total en bloc spondylectomy, with a literature review focusing on long-term recurrence. A 52-year-old male with a two-month history of numbness in the bilateral lower extremities was referred to our hospital. Imaging studies showed a tumor originating in the T9 vertebra and extending to the T8 and T10 vertebrae, with extraosseous extension causing spinal-cord compression. Ten months after onset, the patient presented with progressive paraparesis and hypalgesia. Total en bloc spondylectomy was performed, and pathology was consistent with cavernous hemangioma. Motor and sensory deficits improved significantly, and no signs of recurrence are seen at 2.5 years after operation. A review of literature revealed a recurrence rate of 12.7% (10/79 cases). The available evidence indicates satisfactory long-term outcomes for total tumor resection without adjuvant radiotherapy.

## 1. Introduction

Vertebral hemangiomas are relatively common, found in 10–12% of spines at autopsy [1, 2]. However, symptomatic vertebral hemangiomas are rare, comprising only 0.9–1.2% of all vertebral hemangiomas [2, 3]. Hemangiomas can compress the spinal cord through several mechanisms, including (1) extraosseous extension of the tumor into the extradural space, (2) vertebral body enlargement, (3) compression fracture of an involved vertebra, and (4) extradural hematoma secondary to the tumor [1, 4].

Vertebral hemangiomas with extraosseous extensions causing neurological deficits are locally aggressive, and their prognosis and treatment should be considered separately from other vertebral hemangiomas. Various treatments have been used alone or in combination in recent years—percutaneous sclerotherapy, vertebroplasty, decompressive surgery without tumor resection, subtotal or total tumor resection, radiotherapy, and arterial embolization—but the best treatment for this type of hemangioma remains controversial [1, 5–7]. Although the average age reported for patients diagnosed with symptomatic hemangiomas ranges from 43 to 52 years, there are few reports on long-term clinical outcomes or tumor recurrence rates [1, 6]. We report a case of vertebral hemangioma with extraosseous extension causing progressive paraparesis, treated by total tumor resection.

## 2. Case Presentation

A 52-year-old Asian male with no relevant past or family history presented with a two-month history of numbness in the bilateral lower extremities. The patient was referred to our hospital after computed tomography (CT) and magnetic resonance imaging (MRI) revealed a tumor in the thoracic spine. A neurological examination at the initial visit did not reveal any obvious muscle weakness or sensory deficit. Although the deep tendon reflexes in the lower extremities were exaggerated, no pathologic reflex was observed. The Japanese Orthopaedic Association (JOA) score for thoracic myelopathy was 10/11. A CT-guided biopsy was performed but was inconclusive. An open biopsy was subsequently performed; however since the tumor bled profusely only a small sample could be obtained. Pathological examination revealed that there were increased vessels, which were immune-positive for CD31, CD34, and factor VIII and negative for CK7, CK20, AE1/AE3, CAM5.2, 34*β*E12, S100, and HMB45 (data not shown); however the specimen was too small for a conclusive diagnosis. These findings were consistent with a vascular tumor, and vertebral hemangioma was considered most likely from the tumor site and radiographic findings. Since the patient's numbness subsided and malignant vascular tumors of bone are very rare, further treatment was not performed at this time. The patient was referred due to relocation and was lost to follow-up.

**Figure 1:**
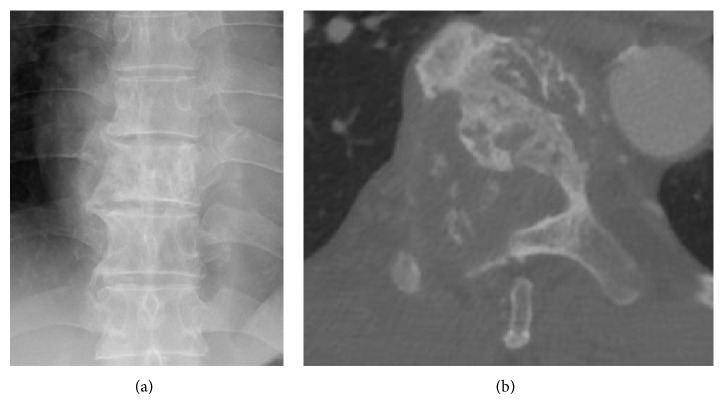
Preoperative images showing a vertebral tumor destroying the T9 vertebra and right pedicle. (a) Preoperative anteroposterior radiograph, (b) CT of the T9 vertebra.

**Figure 2:**
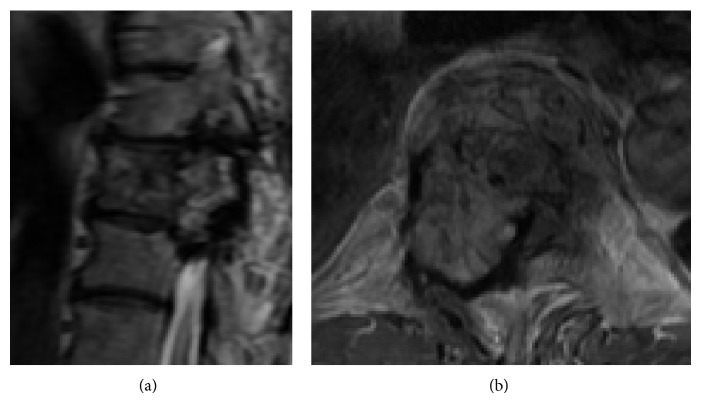
Preoperative MRI showing a multilevel invasive vertebral tumor with extraosseous extension. (a) Sagittal T2-WI, (b) axial gadolinium-DTPA-enhanced T1-WI image (T9).

Ten months after the onset of symptoms, the patient presented with progressive paraparesis and hypalgesia below the T10 dermatome level. Lower-extremity muscle strength was 4/5 bilaterally, and the patient could not climb stairs without support. Bladder function was intact. JOA score for thoracic myelopathy declined to 7.5/11. The right T9 pedicle showed a winking owl sign on anteroposterior radiographs of the thoracic spine, and CT scans showed a tumor destroying the T9 vertebral body and right pedicle (Figure 1). MRI revealed a tumor originating in the T9 vertebra and extending to the T8 and T10 vertebrae (Figure 2). The tumor showed low- to isosignal intensity on T1-weighted images (WI), iso- to high-signal intensity on T2-WI, and heterogeneous enhancement on gadolinium-DTPA-enhanced T1-WI. The extraosseous tumor lesion extended into the spinal canal, with significant compression of the spinal cord. There was no apparent difference between the radiographic findings at the first visit and at the onset of progressive paraparesis. The patient's rapid neurological decline warranted immediate treatment, and since the biopsy was inconclusive and the tumor extended both extraosseously and to adjacent vertebrae, total resection of the tumor by total en bloc spondylectomy (TES) was chosen.

**Figure 3:**
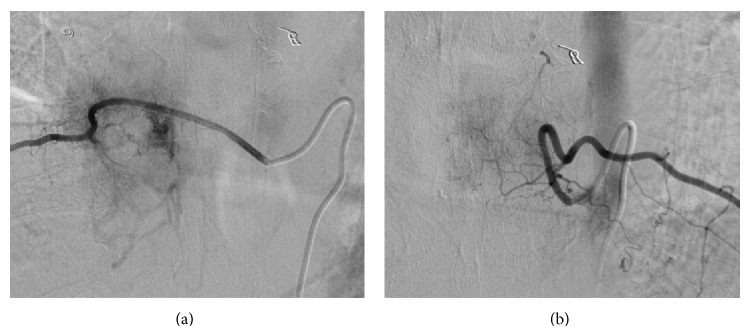
Preoperative spinal angiogram showing a tumor blush. T9 intercostal arteries: (a) right, (b) left.

**Figure 4:**
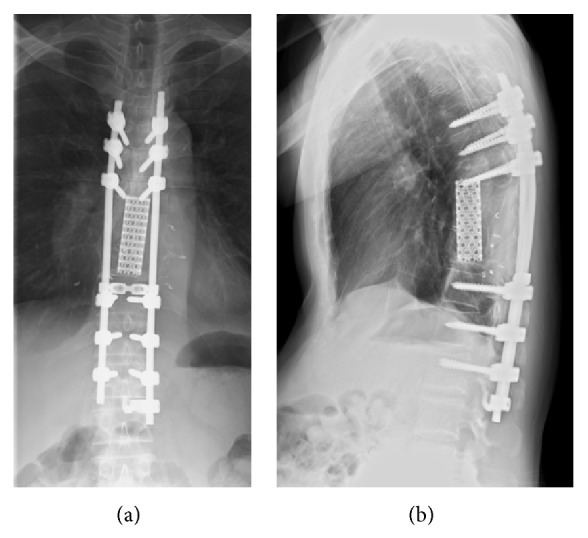
Postoperative radiographs. (a) Anteroposterior, (b) lateral.

**Figure 5:**
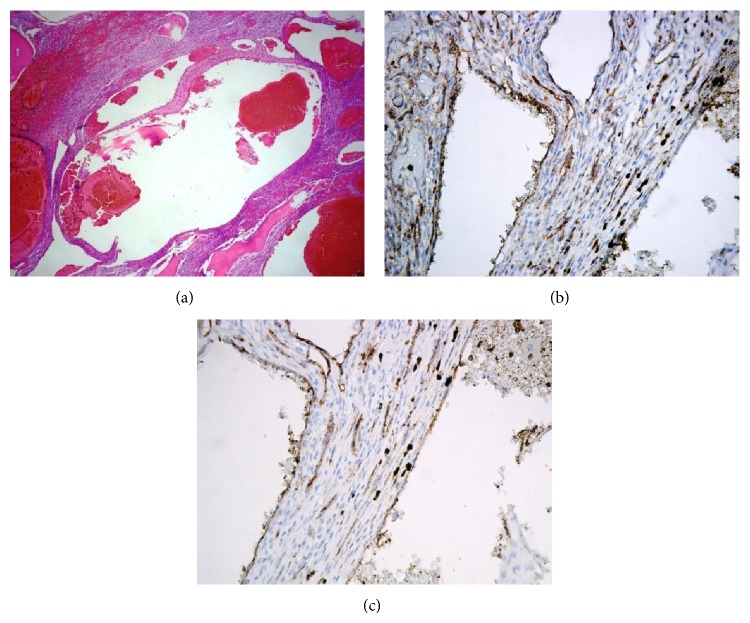
Immunohistological staining of the surgical specimen. (a) Staining of hematoxylin and eosin (×100), (b) CD31 (×400), and (c) CD34 (×400).

Arterial embolization was performed prior to surgery to reduce intraoperative bleeding. A spinal angiogram showed a tumor blush with enhancement of the bilateral T9 intercostal arteries, and a three-level arterial embolization of the bilateral T8 to T10 intercostal arteries was performed (Figure 3). On the next day, the tumor was resected en bloc by TES of the T8, T9, and the cranial half of the T10 vertebrae. The spine was reconstructed with anterior structural support, consisting of a titanium mesh cage packed with minced rib uncontaminated by the tumor, and posterior instrumentation, provided by a pedicle-screw system (CD Horizon Legacy, Medtronic, Memphis, TN) with screws placed in the T5 to L1 pedicles (Figure 4). The surgery took 413 minutes, and the estimated blood loss was 2232 g. Histologically, the surgically resected specimen was composed of a proliferation of irregularly sized vessels and stromal cells; immunohistologically, the cells lining the vessels were positive for CD31, CD34, and factor VIII (Figure 5). Based on these findings, the tumor was diagnosed as a cavernous hemangioma.

**Figure 6:**
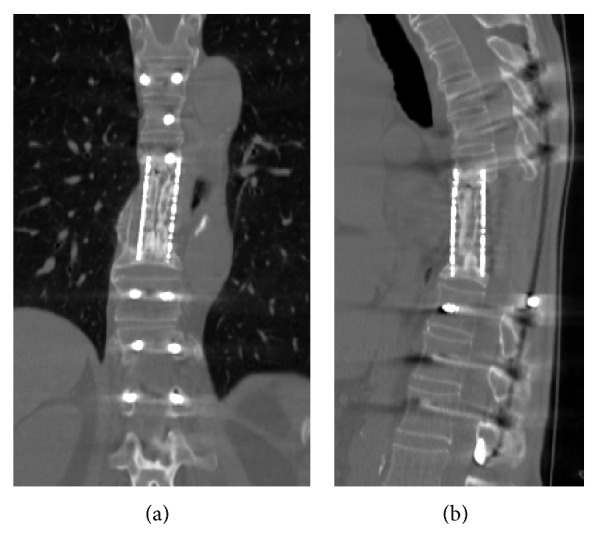
CT scans at 20 months after operation showing bone union. (a) Coronal, (b) sagittal.

The patient's motor and sensory deficits improved significantly, and he was discharged 18 days after surgery without any perioperative complications. He did not receive postoperative radiotherapy. Bone union without local tumor recurrence was confirmed at 20 months after operation by CT (Figure 6). At the last follow-up appointment, 2.5 years after surgery, the patient's JOA score for thoracic myelopathy was 11/11. Informed consent was obtained from the patient for publication of this case report.

## 3. Discussion

The treatment of symptomatic vertebral hemangiomas with extraosseous extensions causing neurological deficits poses a challenge. Treatment options include radiotherapy, arterial embolization, percutaneous sclerotherapy, vertebroplasty, surgical decompression, subtotal tumor resection, total tumor resection, or a combination of these treatments.

Vertebral hemangiomas are radiosensitive; the lesions have been shown to respond to low-dose radiation (30–40 Gy). Although radiation therapy is the most common treatment for lesions that cause pain [6], its use as a sole therapy for patients with progressive neurological deficits is controversial. Most authors have favored surgical decompression, with radiotherapy often used as an adjuvant [6].

Arterial embolization has been reported to reduce intraoperative bleeding and to reverse neurological deficits in some cases [8]. In cases with progressive neurological deficits, arterial embolization is mainly performed preoperatively, to reduce intraoperative bleeding [1].

Recent studies have reported excellent neurological recovery after percutaneous sclerotherapy with injections of either absolute ethanol or 5% ethanolamine oleate [7, 9–12]. This procedure was first reported in 1994 by Heiss et al. in the treatment of two patients with progressive paraparesis due to the extraosseous extension of a vertebral hemangioma [13]. Goyal et al. reported neurological improvement in 85% of patients given percutaneous sclerotherapy [7], and Gabal reported observing neurological improvements within two weeks in five cases of vertebral hemangiomas with extraosseous extension [9]. In Gabal's report, follow-up imaging showed no regression of the epidural portion of the tumor one week after the procedure, but regression was observed at two months [9]. Notably, vertebral collapse is a potential complication with this procedure, reportedly occurring in almost 20% of the patients receiving it [7, 12]. In addition, percutaneous sclerotherapy has two significant limitations: there are insufficient data regarding long-term recurrence after this procedure, and only one case report has reported the use of percutaneous sclerotherapy combined with vertebroplasty for treatment of multilevel lesions [7, 9–12, 14].

Surgical subtotal tumor resection combined with postoperative radiation has been the therapy of choice for many years, because total tumor resection is associated with a high intraoperative morbidity due to massive hemorrhage. However, local recurrence of the tumor after subtotal resection has been reported, and adjuvant radiotherapy makes a second surgery difficult [6]. Therefore, radical surgical resection has been advocated for hemangiomas with an extraosseous extension causing neurological symptoms [3].

Kato et al. reported five cases of aggressive hemangiomas treated with preoperative arterial embolization and surgical total tumor resection by TES; the mean intraoperative blood loss was 2424 mL, and the mean operative time was 608 minutes [3]. There were no perioperative complications, and even without postoperative radiation therapy none of the patients experienced a recurrence of the tumor during a mean follow-up period of 135.2 months [3]. In a retrospective review of 79 patients with spinal tumors treated with TES, Murakami et al. found that none of the patients showed neurological deterioration and concluded that TES is safe with respect to spinal-cord blood flow [15]. Compared to piecemeal excision, TES reduces blood loss and minimizes the risk of residual tumor or tumor contamination, theoretically resulting in lower rates of long-term recurrence.

**Figure 7:**
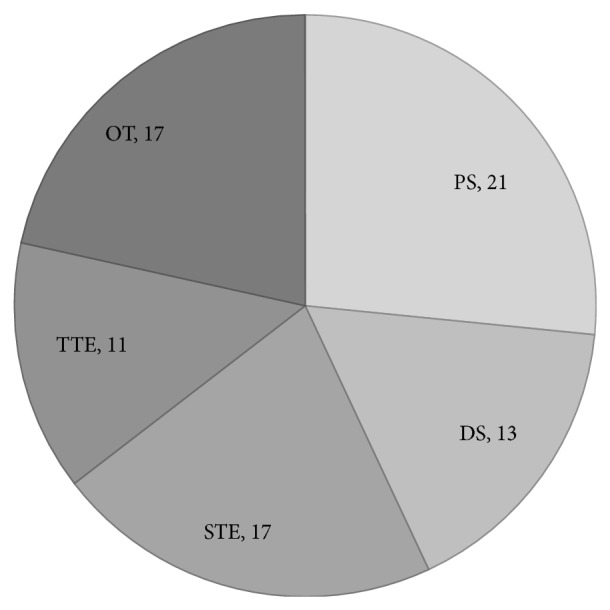
Cases of vertebral hemangioma with extraosseous extension causing neurological deficits in previous reports. PS, percutaneous sclerotherapy; DS, decompression surgery; STE, subtotal tumor excision; TTE, total tumor excision; OT, other treatments.

We reviewed previously reported cases of vertebral hemangioma and identified 79 cases with extraosseous extension causing neurological deficits. Of these, 21 cases were treated by percutaneous sclerotherapy, 13 by decompression surgery without tumor resection, 17 by subtotal tumor resection, 11 by total tumor resection, and 17 by other treatments or a combination of treatments (Figure 7) [1–3, 5–7, 9, 11, 13, 14, 16–31]. Adjuvant radiotherapy was performed in 16 cases and preoperative arterial embolization in 19. The tumor recurred in 10 of the 79 (12.7%) cases (Table 1) [2, 6, 7, 17, 23, 25].

In our present case, the tumor extended both extraosseously and to adjacent vertebrae, and the patient's rapid neurological decline warranted immediate treatment. Percutaneous sclerotherapy was not chosen since the results of this method are still unclear for multilevel lesions and because of the high risk of vertebral collapse due to destruction of the vertebra by the tumor. We selected total tumor resection rather than subtotal tumor resection with adjuvant radiotherapy, because of the lower risk of long-term recurrence. Kato et al. reported two cases of TES treatment of aggressive vertebral hemangiomas that extended to adjacent vertebrae [3]. In agreement with our case, they found that this procedure was associated with reduced intraoperative bleeding and a low risk of recurrence for tumors extending beyond the vertebra [3].

Although the treatment of locally aggressive vertebral hemangiomas is still controversial, the available evidence indicates satisfactory long-term outcomes for total tumor resection without adjuvant radiotherapy. Reports on the long-term outcomes of these tumors are limited, and further studies are needed.

## Figures and Tables

**Table 1:** Cases of hemangioma recurring after initial treatment.

Authors	Initial treatment	Time to recurrence (y)	Additional treatment
Nguyen et al. [2]	STE	1	AE + RT

	DS + AE	6	TTE
Fox and Onofrio [6]	DS + RT	17	RT
	STE	5	DS + RT

Kiroglu et al. [25]	AE	2	VP + SF

Goyal et al. [7]	PS	0.1	DS + AE

Cherian et al. [17]	STE	3	RT

	STE	1.2	DS + RT
Jiang et al. [23]	DS	1	DS + RT
	DS	9	RT + DS + VP

AE, arterial embolization; DS, decompression surgery; PS, percutaneous sclerotherapy; RT, radiotherapy; SF, segmental fixation; STE, subtotal tumor excision; TTE, total tumor excision; VP, vertebroplasty.
